# SARS-CoV-2 outbreak in a synagogue community: longevity and strength of anti-SARS-CoV-2 IgG responses

**DOI:** 10.1017/S0950268821001369

**Published:** 2021-06-24

**Authors:** Yael Gozlan, Stephen Reingold, Ravit Koren, Osnat Halpern, Gili Regev-Yochay, Carmit Cohen, Asaf Biber, Orit Picard, Ella Mendelson, Yaniv Lustig, Orna Mor

**Affiliations:** 1 Central Virology Laboratory, Ministry of Health, Israel; 2 Meuhedet Health Maintenance Organization, Modiin, Israel; 3 Infection Prevention & Control Unit, Sheba Medical Center affiliated to Tel-Aviv University, Tel Hashomer, Israel; 4 Gastroenterology Laboratory, Sheba Medical Center, Ramat-Gan, Israel; 5 Sackler Faculty of Medicine, School of Public Health, Tel-Aviv University, Tel Aviv, Israel

**Keywords:** Herd immunity, IgG antibodies, neutralising antibodies, SARS-CoV-2, sero-prevalence

## Abstract

Severe acute respiratory syndrome-coronavirus-2 (SARS-CoV-2) pandemic is still ongoing along with the global vaccination efforts against it. Here, we aimed to understand the longevity and strength of anti-SARS-CoV-2 IgG responses in a small community (*n* = 283) six months following local SARS-COV-2 outbreak in March 2020. Three serological assays were compared and neutralisation capability was also determined. Overall 16.6% (47/283) of the participants were seropositive and 89.4% (42/47) of the IgG positives had neutralising antibodies. Most of the symptomatic individuals confirmed as polymerase chain reaction (PCR) positive during the outbreak were seropositive (30/32, 93.8%) and 33.3% of the individuals who quarantined with a PCR confirmed patient had antibodies. Serological assays comparison revealed that Architect (Abbott) targeting the N protein LIASON^®^ (DiaSorin) targeting the S protein and enzyme-linked immunosorbent assay (ELISA) targeting receptor binding domain detected 9.5% (27/283), 17.3% (49/283) and 17% (48/283), respectively, as IgG positives. The latter two assays highly agreed (kappa = 0.89) between each other. In addition, 95%, (19/20, by ELISA) and 90.9% (20/22, with LIASON) and only 71.4% (15/21, by Architect) of individuals that were seropositive in May 2020 were found positive also in September. The unexpected low rate of overall immunity indicates the absence of un-noticed, asymptomatic infections. Lack of overall high correlation between the assays is attributed mainly to target-mediated antibody responses and suggests that using a single serological assay may be misleading.

## Introduction

Seropositivity combined with neutralising capability of IgG antibodies is the ultimate humoral measure of the immune system against severe acute respiratory syndrome coronavirus-2 (SARS-CoV-2) transmission. With the initiation of vaccination efforts, understanding the efficacy and persistence of natural immunity is critical when deciding how to allocate a limited number of vaccines, as well as determining when herd immunity is achieved. Yet, data on the duration of immune responses following natural infection, the persistence of IgG antibodies and their viral neutralising capacity, is still limited.

Seroconversion, determined by positive IgG result in serological assays, occurs usually within 7–14 days following diagnosis. Asymptomatic individuals are considered to have a weaker immune response to SARS-CoV-2 infection compared to those who were clinically ill [1]. However, the durability of IgG response over time is not clear yet; some groups have reported reduction in IgG and neutralising antibody levels in the early convalescent phase [1, 2], whereas others [3] showed that IgG remained stable in the convalescent phase for at least 31 weeks [4]. The different sensitivities and specificities of the serological assays used in these studies, may explain these contradictory reports. Indeed, since a high positive predictive value could not currently be assured using a single test, CDC recommended to use an orthogonal testing algorithm and confirm a positive first assay result with a secondary assay [5]. Virus neutralisation assays which are usually not applicable for high-throughput screening, remain the gold standard approach for determining antibody efficacy and their results could confirm serological assay results. However, it is important to keep in mind that because neutralisations are functional assays they are often less sensitive than other serological tests which only measure the existence of specific antibodies regardless of their neutralisation capacity. Neutralisation assays that utilise SARS-CoV-2 require BSL3 conditions and are therefore difficult to use [6]. Recently, tests which use recombinant pseudoviruses that incorporate the S protein of SARS-CoV-2 and could be used under regular laboratory conditions were demonstrated to efficiently identify neutralising antibodies in patient samples [7].

Two of the first commercial assays approved in April 2020 via the emergency use authorisation (EUA) are the Architect SARS-CoV-2 IgG (Abbott Laboratories) detecting IgG antibodies against the viral N antigen, and the LIASON^®^ SARS-CoV-2 S1/S2 IgG (DiaSorin) identifying IgG antibodies against the viral S1 and S2 proteins. While S protein is a surface protein essential for viral entry, the N protein is a structural protein that binds to the coronavirus RNA genome and is known to be the most abundantly expressed protein of the SARS-CoV-2 [8]. Recently, we have reported 84.7% sensitivity and 99.5% specificity with Architect and 82.4% sensitivity and 98.7% specificity with LIASON for the detection of anti-SARS-CoV-2 antibodies [9]. In addition, using a new receptor binding domain-enzyme-linked immunosorbent assay (RBD-ELISA) identifying IgG antibodies against the viral RBDwithin the S protein, we determined 88% sensitivity and 98% specificity [10].

One of the earliest local outbreaks in Israel occurred in the centrally located city of Modiin, within a synagogue congregation of approximately 600 individuals. The source of the exposure was eventually identified as an individual, who was infected by a returning traveller from the United States. It should be noted that during the time of this outbreak, national health policy did not require any social distancing measures, and that polymerase chain reaction (PCR) testing was not available per request.

Here, we aim to describe the immunity among this outbreak participants and to understand the longevity and strength of the anti-SARS-CoV-2 IgG responses using different serological assays from samples taken 6 months following the exposure. Together with the progress of worldwide anti-SARS-CoV-2 vaccination efforts, these data, among similar studies, are highly important.

## Methods

### Study participants

All members of the Hoshen synagogue community (approximately 600 individuals, adults and children) that were potentially exposed to SARS-CoV-2 outbreak in mid-March 2020, were approached 180 days later, in September 2020. Two hundred and eighty-three individuals agreed to participate in the study. An explanation of the methods and aims of the study and a link to an online questionnaire were provided. Demographics, data on exposure to coronavirus disease-2019 (COVID-19)-positive individuals during and after the local outbreak, symptoms consistent with COVID-19, quarantine (yes/no), SARS-CoV-2 PCR testing results, previous SARS-CoV-2 IgG antibody results and pre-existing medical conditions were requested. Clinical symptoms − fever, cough, throat pain, dyspnoea, anosmia/ageusia, other respiratory symptoms and other constitutional symptoms (malaise, myalgia, headache and gastrointestinal symptoms) were recorded.

This study was approved by the Israeli Ministry of Health and individuals were exempted from signing an informed consent.

### Sample collection and assessment of anti-SARS-CoV-2 antibodies

Blood samples from 283 individuals that agreed to participate in this study were collected in September 2020, six months after the local outbreak. Before this study was initiated, and upon request of the family members, blood samples from 33 of the study participants who were PCR positive or quarantined with a positive PCR patient during the outbreak, were also collected in May 2020 (two months following exposure). Each sample was tested once with the following assays: Architect anti-SARS-CoV-2 IgG test (Abbott Laboratories, Abbott Park, IL), LIASON IgG test (DiaSorin, Centralino, Italy) and a RBD-ELISA. The commercial assays were performed according to the instructions using the cutoff values reported by the manufacturers's (Architect <1.40 is considered negative; LIASON < 12.0 is negative, 12.0–15.0 is equivocal and >15.0 is positive). The RBD- ELISA assay was performed as previously described [10] and the results were interpreted based on the CDC recommendations [5], for a population with a SARS-CoV-2 prevalence of 5%, whereby all results under index value of 1.83 were considered negative [9].

All samples from IgG positive individuals were tested for the presence of viral neutralising antibodies (psNUT). A green fluorescent protein (GFP) reporter-based pseudotyped virus neutralisation assay with a non-replicative vesicular stomatitis virus (VSV) backbone coated with SARS-CoV-2 spike (S) protein was generously obtained from Dr Gert Zimmer (Institute of Virology and Immunology, Mittelhäusern, Switzerland). psNUT assay was technically performed as described [11]. Sera not capable of reducing viral replication by 50% at 1/16 dilution were considered non-neutralising [12, 13].

### Results interpretation

A person was determined seropositive if a positive IgG result was obtained in at least two of the three serological tests. However, for individuals found previously to be SARS-CoV-2 positive by PCR, a single positive antibody test result together with the positive PCR assay results were sufficient to determine positive sero-status. Equivocal results (observed in LIASON test only) were considered positive when either Architect or RBD-ELISA were positive for the same participant.

BioVenn [14] was used to compare the positive IgG results of the different assays and was visualised using area-proportional Venn diagram. The agreement between any two serological assays was assessed using the Cohen's kappa (*κ*) statistic.

## Results

### Outbreak description and characteristics of study participants

The outbreak occurred during the week of Purim, a Jewish holiday, 6–14 March 2020. The index patient was infected by a returning traveller from the United States. Exposures in the community occurred at the synagogue, at individuals' homes, as well as during youth group events. Table 1 summarises basic information on the 283 study participants, members of the Hoshen synagogue community that agreed to participate in this study. More males were included (male/female 1.24); median age was 37 years, (IQR 16–47). Although the majority of all community members (82.7%) were asymptomatic, most of them (89%) were put into quarantine. Seventy of the 283 individuals that agreed to participate were tested by PCR at the time of the outbreak; 36 of them were found positive. The proportion of these confirmed PCR positive individuals was similar in both sexes; 6.7% of children below the age of 10 were PCR positive, 10.8% of those between 11 and 19 years and 14% of adults >19 years were PCR positive.

**Table 1.** Demographic, epidemiological, medical status and SARS-CoV-2 RT-PCR results of study participants (*n* = 283)

Parameter		*n* (%)	PCR positive result, *n* (%)
Sex	Male	157 (55.5)	19 (12.1)
	Female	126 (44.5)	17 (13.5)
Age	0–10	15 (5.3)	1 (6.7)
	11–19	83 (29.3)	9 (10.8)
	>19	185 (65.4)	26 (14)
Symptoms	Any	49 (17.3)	32 (65.3)
	None	234 (82.7)	4 (1.7)
Quarantine	Yes	254 (89.8)	36 (14.2)
	No	29 (10.2)	0
Pre-existing medical conditions*	Any	56 (19.8)	8 (14.3)
	None	227 (80.2)	28 (12.3)

*Preexisting medical conditions included one of the followings: diabetes, obesity, cancer, hypertension, cardiac disease, pulmonary disease, hepatic disease, immunologic disorders, solid organ or bone marrow transplantation, or immune suppression treatment.

### Comparison between IgG and PCR results

**Fig. 1.**
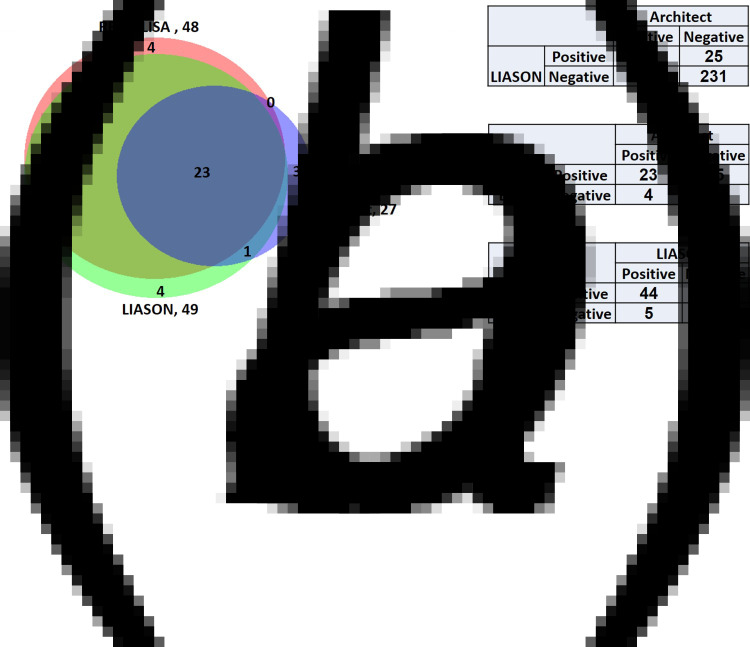
Comparison between the serological assays used in this study. (a) Venn diagram representing sero-positivity of the different assays. (b) 2 × 2 tables of the three serological assays. Presented are the number of patients positive or negative for any two assays. Kappa values were: 0.58, 0.56, and 0.89 for Architect and LIASON, Architect and RBD-ELISA and LIASON and RBD-ELISA, respectively.

The total number of IgG positive samples was 27, 48 and 49 for the Architect, RBD-ELISA and LIASON, respectively. LIASON and RBD-ELISA shared the highest number of positive results as observed by the Venn diagram (Fig. 1a). Indeed, the highest agreement between assays (kappa = 0.89) was obtained for LIASON and the RBD-ELISA assays. Moderate agreement was found between Architect and LIASON (kappa = 0.58) and between Architect and RBD-ELISA (kappa = 0.56, Fig. 1b).

**Fig. 2.**
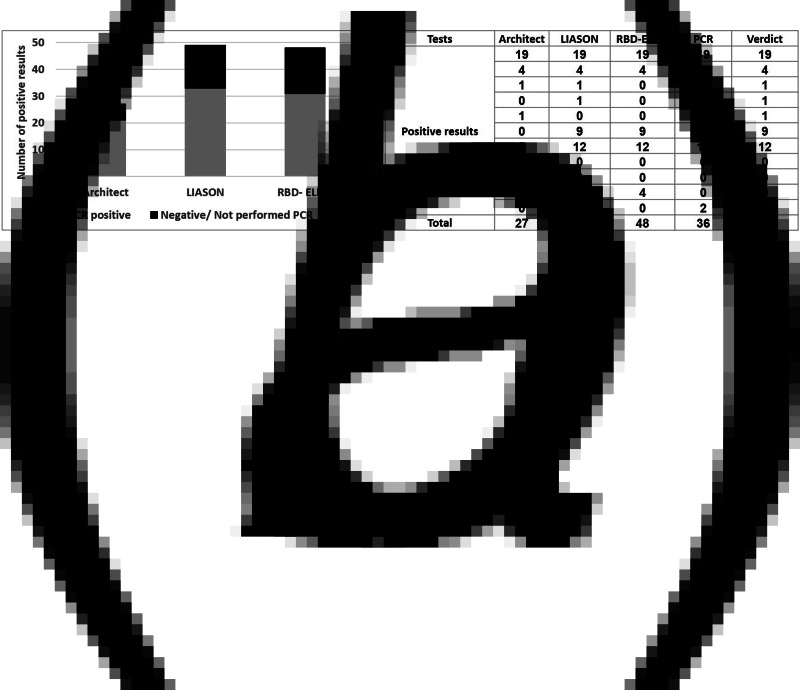
Segregation of positive results between serological and PCR assays. (a) The cumulative number of PCR positive and negative results in each serological assay. (b) Serological verdict based on the results of the serological and PCR assays.

When PCR results were also considered, the proportion of those confirmed to be positive by PCR among those with positive IgG results in each of the assays was between 65% and 77% (Fig. 2a). Finally, for an individual to be seropositive, at least two assays had to be positive as described in methods section. Accordingly, 47 individuals were hereby considered to be positive for anti-SARS-Cov-2 IgG antibodies (Fig. 2b). As only 36 individuals were confirmed as PCR positive during the outbreak, the likely proportion of unrecognised infections was 23.4% (11/47). All individual IgG, PCR and final serological verdict is presented in Supplementary Figure S1.

### Correlation between antibody responses, PCR results, clinical and epidemiological data

The presence of anti-SARS-CoV-2 IgG antibodies and viral neutralisation capability classified by clinical and epidemiological data is summarised in Table 2. Overall, 89.4% (42/47) of the participants who were considered to have positive IgG results (47/283, 16.6%) had neutralising antibodies.

**Table 2.** Serological (IgG antibodies) and neutralization (psNUT) results classified by clinical and epidemiological data. psNUT analysis was performed only on samples with positive IgG results

			Positive IgG *n* (%)	psNUT *n* (%)
Age (years, y)	*n* = 283	0–10 y, *n* = 15	2 (13.3)	2 (100)
		11–19 y, *n* = 83	15 (18.1)	13 (86.7)
		>19 y, *n* = 185	30 (16.2)	27 (90)
Symptoms	Any *n* = 49	Positive PCR, *n* = 32	30 (93.8)	28 (93.3)
		Negative PCR, *n* = 8	6 (75)	5 (83.3)
		Not tested/no result PCR, *n* = 9	1 (11.1)	0
	None *n* = 234	Positive PCR, *n* = 4	4 (100)	4 (100)
		Negative PCR, *n* = 67	2 (3)	1 (50)
		Not tested/no result PCR, *n* = 163	4 (2.5)	4 (100)
Quarantine*	*n* = 247	With a confirmed patient, *n* = 15	5 (33.3)	3 (60)
		Without a confirmed patient, *n* = 203	9 (4.4)	8 (88.9)
		No, *n* = 29	0	0
Pre-existing medical conditions	*n* = 283	Yes, *n* = 56	9 (16.1)	8 (88.9)
		No, *n* = 227	38 (16.7)	34 (89.5)

*not including confirmed patients.

More symptomatic individuals with PCR positive results were considered seropositive (30/32, 93.8%) compared to those who did not have positive PCR results (7/17, 41.2%). In addition, more individuals who quarantined with a PCR confirmed patient had antibodies (5/15, 33.3%) compared to those who quarantined with none-PCR-confirmed patient (9/203, 4.4%). Positive antibody results were obtained both for young children 0–10 years old (2/15, 13.3%) and teenagers, 11–19 years old (15/83, 18.1%). Also, 16.2% of those >19 years of age were IgG positive.

### Antibody durability

**Fig. 3.**
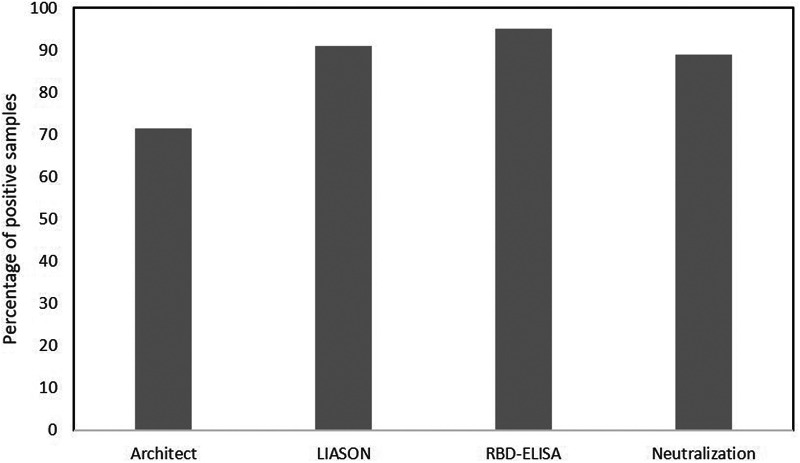
Longevity of IgG 6 months compared to 2 months following the outbreak. Presented in the % of positive results in samples taken 6 months versus 2 months following exposure as detected by the different assays.

Thirty-three of the participants had antibodies tested in blood drawn during May 2020, two months after the outbreak, 23 of which were found IgG positive. This serology evaluation early after the infection, was performed for these participants upon their request, as they were all either PCR positive or quarantined with a confirmed patient. Figure 3 compares IgG results in samples taken two and six months following exposure. Loss of antibodies was mainly observed with Architect assay, whereby 71.4% (15/21) of participants remained IgG positive. RBD-ELISA and LIASON results were positive for 95% (19/20) and 90.9% (20/22) of the samples, suggesting longer durability of these antibodies compared to the Architect. Importantly, neutralising antibodies persisted in 88.9% (16/18) of the participants.

## Discussion

This study assessed persistence of anti-SARS-CoV-2 antibodies in a small and close community exposed to infection in an early time following the start of this global pandemic. Although most individuals were exposed to the virus and many were quarantined, the overall sero-prevalence identified was 16.6%. In a nation-wide screening performed by the Ministry of Health at the same time a sero-prevalence of 5.5% was found. Similarly, during the same period (July–September 2020) low sero-prevalence of 3.1%–5% was reported in several other countries [15–17]. Although the seropositivty of this community was higher than in the general population, it was lower than initially expected. This may result from low assay sensitivity or from short durability of antibodies to this infection. On the other hand, it may suggest that although exposed, most community members were indeed not infected. As could be expected, most of the symptomatic individuals with PCR positive results were seropositive (30/32, 93.8%). In addition, 33.3% of the individuals who quarantined with a PCR confirmed patient had antibodies.

To correctly determine seropositivity, three serological assays were compared and PCR data were also collected. We have shown that the degree of agreement between any two serological assays was different and that the Architect test failed to identify nearly 50% of the IgG positive individuals and was significantly less sensitive compared to the LIASON and the RBD-ELISA which were highly comparable (kappa = 0.89). Moreover, our results demonstrate that persistence of antibodies directed to the viral nucleocapsid (N), the target identified by the Architect test, is inferior to that of the spike protein S1/S2, targeted by LIASON or the RBD. This conclusion is based on the reduced longevity of IgG antibodies detected by the Architect assay, exemplified by the lower prevalence of IgG positive results in samples taken six months compared to those taken two months following the SARS-CoV-2 exposure. Higher durability was observed for antibodies detected by LIASON and RBD-ELISA (90.9% and 95% durability in samples taken in May and in September 2020, respectively). Similar observations were recently reported by others [18]. Indeed, it was already demonstrated that the antibody response to various viral proteins, including S, S1, S2, RBD and N, varies and that assay sensitivity correlates with the abundance, conservation and antigenicity of the different viral proteins, as well as with the durability of the individual antibody response [19, 20].

Most of the seropositive individuals were capable to neutralise SARS-CoV-2 pseudo-virus infection. This result is clinically important and may suggest long-lasting immunity against such viral infection.

In this study, the RBD ELISA test-positive cutoff was calculated to give a sensitivity of 85% and specificity of 99.76%, according to the CDC recommendations, for a population with a SARS-CoV-2 prevalence of 5% [5, 9, 10]. However, our test results were slightly less accurate (86.1% and 97.1%, respectively). The latter may be due to the higher seroprevalence of the study population when compared to the national average for which the cutoff was created.

SARS-CoV-2 is highly infective during incubation period with rapid transmission in teenagers and children. Fast onset and various non-specific atypical manifestations were reported in children [21]. Here, 35% of our cohort were below 19 years of age, and many of them participated in the youth events. However, no significant differences were found between the serological status of the young, below 19 years and those >19 years of age (17.3% and 16.2%, respectively). Thus, it seems that overall immunity in such exposed community, even among the young population does not occur.

Despite being a small, volunteer-based, cohort, there are several advantages to this study. First, the relatively early occurrence of the outbreak in a close community allows for the investigation of antibody durability over six months. Moreover, previous serological results for a sub-cohort, allows to learn more about antibody longevity. Finally, personal details provided by the participants allowed to correlate with epidemiological, medical and demographical data.

This study demonstrates the paucity of overall immunity in an exposed population as well as the varying durability of different antibody responses. Therefore, serological investigations should bear in mind the characteristics of the population that is investigated as well as the technical limitations of serological assays and consider a multiple- and diverse test algorithm. Towards the coming global anti-SARS-CoV-2 vaccination, these data, as well as data from other studies are highly important.

## Data Availability

All data supporting the finding of this study are available upon request from the corresponding author.

## Supplementary material

For supplementary material accompanying this paper visit https://doi.org/10.1017/S0950268821001369. click here to view supplementary material
