# Supplementary figures and images for: SARS-CoV-2 outbreak in a synagogue community: longevity and strength of anti-SARS-CoV-2 IgG responses
Source: Epidemiol Infect. 2021 Jun 24;149:e153. doi: 10.1017/S0950268821001369 (PMC8354684; doi:10.1017/S0950268821001369)

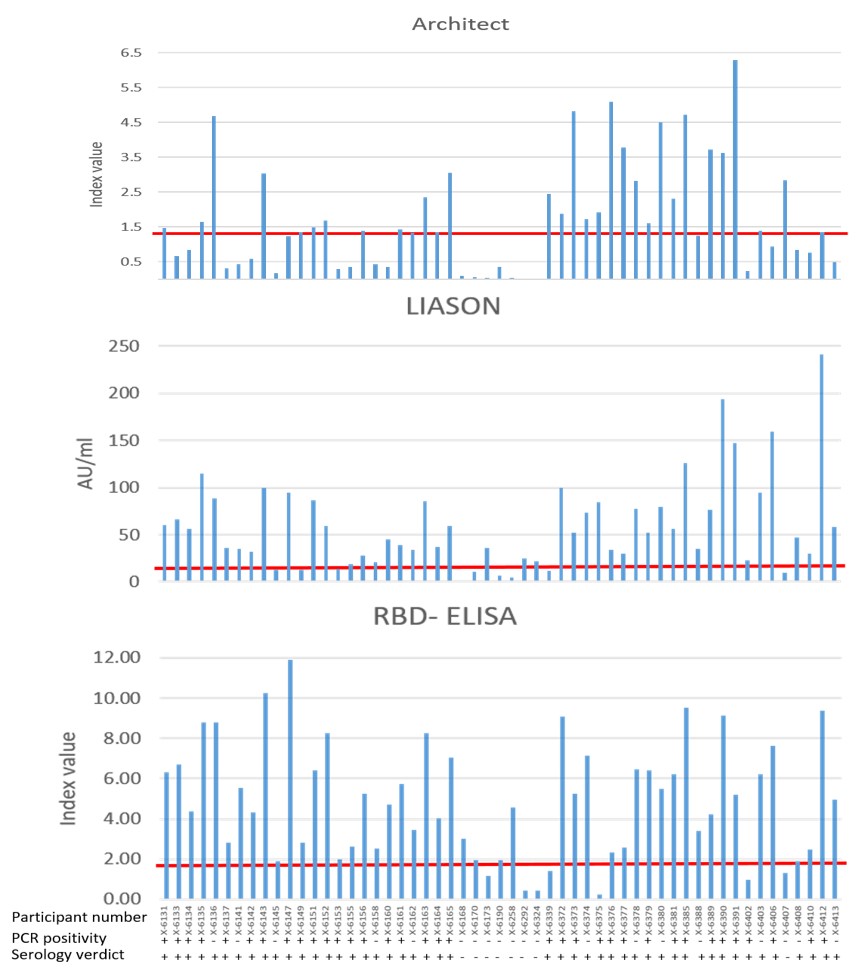

Supplement: Supplementary file 1 [file S0950268821001369sup001.jpg]
